# A new phase for Egyptian Journal of Forensic Sciences, the official journal of The International Association of Law and Forensic Sciences (IALFS)

**DOI:** 10.1186/s41935-017-0014-x

**Published:** 2017-07-18

**Authors:** Magdy Kharoshah, Mohammed Madadin

**Affiliations:** 1 Forensic Medicine Center, Dammam, Kingdom of Saudi Arabia; 2 Dammam University, Dammam, Kingdom of Saudi Arabia

*Egyptian Journal of Forensic Sciences (EJFS)* is an open access journal publishing articles in the forensic sciences, pathology and clinical forensic medicine and its related specialties. The journal publishes classic reviews, case studies, original research, hypotheses and learning points, offering critical analysis and scientific appraisal. (https://ejfs.springeropen.com/).

*EJFS* is the official publication of The International Association of Law and Forensic Sciences (IALFS), and is indexed in Scopus and DOAJ, as well as having a SCImago Journal Rank (SJR) of 0.35 and a Source Normalized Impact per Paper (SNIP) of 0.705. *EJFS* previously published quarterly by Elsevier between January 2011 and December 2016. All issues (volumes 1–6) are available on Science Direct (http://www.sciencedirect.com/science/journal/2090536X). However, we are thrilled to announce that since January 2017, *EJFS* transferred to SpringerOpen, one of the largest and most reputable journal publishers worldwide. The journal will now publish in a continuous format. Our editorial board has also been refreshed for this new phase to include well-recognized editors and scientists in the field from all over the globe (https://ejfs.springeropen.com/about/editorial-board).

Publishing with SpringerOpen is a great opportunity for us as an open access journal. All articles are freely and universally accessible online, so an author’s work is available to readers at no cost, and is not limited by their library’s budget. This ensures that research is disseminated to the widest possible audience, as open access journals have the potential to reach a much larger set of readers than any subscription-based journal, in print and online (http://www.biomedcentral.com/info/about/charter; http://opcit.epr1ints.org/oacitation-biblio.html; Suber, 2005). Studies have suggested a correlation between open access, higher downloads and higher citations, potentially leading to a higher Impact Factor (http://opcit.epr1ints.org/oacitation-biblio.html; Suber, 2005; http://eprints.ecs.soton.ac.uk/10713/02/timcorr.htm).

Furthermore, publication costs for *EJFS* are covered by the Specialized Presidential Council for Education and Scientific Research (Government of Egypt), so authors do not need to pay an Article Processing Charge (APC). This is important to us to ensure a country’s economy will not influence its researchers’ ability to access articles, regardless if a submitting institution is resource-rich or poor. By having covered open access APCs, we aim to utilize all access to the internet to its full advantage to increase the visibility of research, especially from developing countries where this may be limited (Tan-Torres Edejer, 2000).

All manuscripts will undergo rigorous peer-review prior to publication. EJFS operates a double-blind peer-review system, where the reviewers do not know the names or affiliations of the authors, and reviewer reports provided to authors are anonymous. Independent researchers in the relevant areas will assess submitted manuscripts for originality, validity, and significance, to help editors determine whether the manuscript should be published in the journal (EJFS, https://ejfs.springeropen.com/submission-guidelines/peer-review-policy).

Authors hold copyright for their work and grant anyone the right to reproduce and disseminate the article, provided that it is correctly cited and no errors are introduced.

In the near future, we will be applying for inclusion in PubMed Central and hence PubMed (http://www.pubmedcentral.org), as well as other freely accessible, full-text repositories. This complies with the policies of a number of funding bodies including the Wellcome Trust, NIH and Howard Hughes Medical Institute (http://www.biomedcentral.com/info/about/apcfaq#grants; http://www.nih.gov/news/pr/feb2005/od-03.htm; http://www.wellcome.ac.uk/node3302.html; http://www.hhmi.org/about/research/sc320.pdf). We have worked hard to ensure a smooth transition and begin this next phase of continuous publication as soon as possible. We have been receiving a good number of manuscripts to consider since the launch of our submission system, and we are very keen to maintain our fast peer review process to maintain competitive submission to publication times.

We invite readers and researchers to visit our new website at SpringerOpen, and to submit your work to *EJFS* (https://www.editorialmanager.com/ejfs/default.aspx). As Editor-in-Chief, I would like to acknowledge the Associate Editors, Publisher, Editorial Board Members, referees and authors for their contribution to the journal over the years.
